# Searching for consensus among physicians involved in the management of sick-listed workers in the Belgian health care sector: a qualitative study among practitioners and stakeholders

**DOI:** 10.1186/s12889-016-2696-7

**Published:** 2016-02-17

**Authors:** Marc Vanmeerbeek, Patrick Govers, Nathalie Schippers, Stéphane Rieppi, Katrien Mortelmans, Philippe Mairiaux

**Affiliations:** Department of General Practice/Family Medicine, University of Liege, Quartier Hôpital, Avenue Hippocrate 13, CHU B23, Liege, 4000 Belgium; Department of Occupational Health and Health Promotion, University of Liege, Quartier Hôpital, Avenue Hippocrate 13, CHU B23, Liege, 4000 Belgium; Analyse et évaluation des politiques publiques – Méthodologie de science politique, University of Liege, Quartier Agora, place des Orateurs 3, Bât. B31, Liege, 4000 Belgium; Department Research & Development, Italiëlei 2, Antwerpen, 2000 Belgium

**Keywords:** Cooperative behaviour [MeSH], Attitude of health personnel [MeSH], Delphi technique [MeSH], Sick leave [MeSH]

## Abstract

**Background:**

In Belgium, the management of sick leave involves general practitioners (GPs), occupational health physicians (OPs) and social insurance physicians (SIPs). A dysfunctional relationship among these physicians can impede a patient’s ability to return to work. The objective of this study was to identify ways to improve these physicians’ mutual collaboration.

**Methods:**

Two consensus techniques were successively performed among the three professional groups. Eight nominal groups (NGs) gathered 74 field practitioners, and a two-round Delphi process involved 32 stakeholders.

**Results:**

From the results, it appears that two areas (reciprocal knowledge and evolution of the legal and regulatory framework) are objects of consensus among the three medical group that were surveyed. Information transfer, particularly electronic transfer, was stressed as an important way to improve. The consensual proposals regarding interdisciplinary collaboration indicate specific and practical changes to be implemented when professionals are managing workers who are on sick leave. The collaboration process appeared to be currently more problematic, but the participants correctly identified the need for common training.

**Conclusions:**

The three physician groups all agree regarding several inter-physician collaboration proposals. The study also revealed a latent conflict situation among the analysed professionals that can arise from a lack of mutual recognition.

Practical changes or improvements must be included in an extended framework that involves the different determinants of interdisciplinary collaboration that are shown by theoretical models. Collaboration is a product of the actions and behaviours of various partners, which requires reciprocal knowledge and trust; collaboration also implies political and economic structures that are led by public health authorities.

## Background

In Europe, demographic projections indicate that by 2060, two working-age individuals will be needed to support one pensioner [1]. This rapidly ageing population is thus presenting challenges to the sustainability of social protection schemes. Waddell & Burton demonstrated a strong association between worklessness and poor health, including higher mortality and poorer general and mental health, which result in more medical consultations, greater medication consumption and higher hospital admission rates [2]. In this context, the need for a more active policy towards the professional reintegration of long-term sick-listed workers has prompted the Belgian Federal Public Service (FPS) Employment and Labour to fund a study to investigate the best ways to promote an efficient collaboration among the health professionals who manage sick leave.

Effective collaboration and cooperation among health professionals is nevertheless a challenge. As shown in the literature, various obstacles must be overcome, such as 1) possible issues in interpersonal relationships (e.g., the differences in cultural values concerning teamwork, willingness to collaborate and trust), 2) organisational determinants within the care teams (e.g., hierarchy, administrative support and communication, and collaboration mechanisms), and 3) systemic determinants (e.g., social factors such as gender or social status and trends in professional domination and control) in the health system environment [3–5]. Most studies focus on interprofessional collaboration in acute or chronic care settings, e.g., between physicians and nurses [6, 7]. The studies that examine the collaboration among general practitioners (GP), occupational physicians (OP) and social insurance physicians (SIP) are very rare [8, 9]. In Belgium, Mortelmans reviewed the existing studies on the co-operation practices among physicians, specifically the practices that involved sickness absence, work resumption, and disability management [10]. Co-operation among SIPs, curative physicians, and OPs was rare, although it was desired by most of them [11, 12]. The obstacles to communication were mainly physical (i.e., difficulty in reaching the OP) or practical (i.e., miscommunications concerning the legal context for information exchange); moreover, the respective job content was poorly understood or mistrusted. Curative physicians and SIPs were thought to lack interest in patients’ work conditions [13].

In Belgium, universal health insurance guarantees care for anyone who is ill and/or claims to need medical care. Sickness benefits are paid by the National Institute for Health and Disability Insurance (NIHDI) after 30 days of sick leave; during the first 30 days, the patient’s earnings are paid by the employer. Sick notes are issued by the GP, and their validity is checked by the SIP that is attached to one of the health insurance funds. Based on a patient interview and clinical examination, the SIP may encourage a return to work or, when the patient is not willing to return, may decide to end sickness benefits. When returning to work, the patient/worker may benefit from the intervention of an OP because every enterprise must affiliate with an occupational health service (OHS).

Considering their respective roles in sick leave management, more structured dialogue among GPs, OPs and SIPs can be anticipated to facilitate a return to work at the most appropriate time and, if needed, with adaptations in the work environment. The expert opinion is that this collaboration can help to reduce long-term sick leave, disability and job loss; however, there is little scientific evidence to support these assumptions [9].

This study’s main objective was to find an inter group consensus on the practical means to improve the collaboration among GPs, OPs and SIPs when managing workers who are on sick leave.

## Methods

To seek consensus among these health professionals, the following two methods were combined: the nominal group technique (NGT) and the Delphi method [14, 15]. Both methods are widely used, and the consensus level is similar irrespective of the method that is used [16, 17]. A combination of the two methods is used when relevant, depending on the type of consensus that must be reached and the desired representativeness of the participants [18]. For this study, a group process was desirable in the first phase (the gathering of physician opinions), whereas an anonymous process was needed in the second phase (a consensus search among people who may have conflicting opinions or who are limited in expressiveness in face-to-face meetings).

### Nominal group technique

To allow a bottom-up process, the NGT was used to separately retrieve the GP, OP and SIP opinions and wishes on collaborative work. The participating physicians were field practitioners and thus were considered experts in this particular area of their jobs. These physicians met in single professional groups (Table 1).

**Table 1:** Participants of the nominal groups

Professions		Participants	Total
GPs	Urban practices (2 groups)	7 + 11	42 (8 female)
	Rural practices (2 groups)	13 + 11	
OPs	2 groups	9 + 7	16 (3 female)
SIPs	2 groups	9 + 7	16 (6 female)
			74

The GPs were recruited from existing groups (local peer-review or continuous professional development groups). The OPs were recruited through the internal occupational health services (IOHS) or external OHS (EOHS). The SIPs were members of the technical council of the National Institute for Health and Disability Insurance (NIHDI).

The question addressed to each group was the following: “*Can you remember some specific cases for which you were required to be in contact or collaborate with another health professional, i.e., a GP, an OP or an SIP? Keeping these cases in mind, how do you think you could improve the collaborative work among GPs, OPs and SIPs?”*

The items that resulted from the round robin (the sharing of the ideas that the participants generated) and clarification (the verbal explanation regarding the proposals) phases were ranked by each participant from one to five points according to the priority that they gave each item. The votes were summed and converted to percentages for each physician group.

### Delphi technique

In a second phase, a two-round Delphi process was conducted to test the legitimacy of the proposals that resulted from the NGT phase and to ensure their acceptance among the stakeholders. This phase was conducted among about thirty stakeholders, representatives from the three physician groups. The participants were selected by the respective university departments, physician unions, professional associations and scientific societies. Considerable attention was given to a proper balance among the participant languages, i.e., Dutch and French.

No firm rules have been established in the literature to determine when a consensus is reached, and the authors could choose among various methods [19–21]. In this study, a proposal reached consensus if it received at least 75 % approval among the respondents overall and in each of the physician groups. The survey participants had to answer each proposal by using a five-point Likert scale that ranged from “strongly agree” to “strongly disagree” and included a “not concerned” item, and they were invited to comment in free text. The “agree” and “strongly agree” responses were pooled to calculate the approval rate.

Specialised software, namely, Mesydel®3.0 (Spiral, Laboratory investigation, Political Science and criminology dept., University of Liège, Belgium), was used to conduct the Delphi survey online. This software provides the capacity for the respondents to write comments and for the researchers to tag these answers according to the topics that were addressed, which is a process that was inherited directly from the social/semantic web and its folksonomies. A “tag cloud” is automatically built and provides visual clues of the relative weight of the themes in either the complete corpus or the frame of a given question.

The first Delphi round began in April 2012 and lasted five weeks; the second round started in late June 2012 and remained open for twelve weeks.

### Multidisciplinary approach

A multidisciplinary approach was applied to facilitate the triangulation process when collecting and analysing the data [22–24]. The research team involved experts from both the three physicians groups (GPs, OPs, and SIPs) under study and the researchers from the social sciences and humanities (psychology, anthropology, and sociology). A committee that comprised the stakeholders from the professional bodies and representatives of the Ministry of Employment was established to support the research team.

## Results

### Nominal groups

A total of 74 physicians participated, including 42 GPs (four groups), 16 OPs (two groups) and 16 SIPs (two groups); the allocations to French- and Dutch-speaking groups were similar in each profession (20 compared with 22 GPs, 9 compared with 7 OPs and 9 compared with 7 SIPs) (Table 1).

A total of 124 proposals were generated by the eight groups, and 106 of these proposals were ranked. A qualitative analysis of the items allowed classification into the following four classes: 1) operational information transfer; 2) mutual collaboration; 3) knowledge; and 4) evolution of the legal and regulatory framework. The rankings of the groups that belong to the same physician category were summed and converted to percentages (Table 2).

**Table 2:** Item ranking in nominal groups, by physician category (in %)

	GPs	OPs	SIPs
Making information transfer operational			
Availability of clinical data via electronic exchanges	11.4	16.3	14.1
Conventional communication tools (post, phone)	1.5	0	0
Patient as a communication vector	0.0	28.3	0
Availability of an address and phone book	5.1	6.3	19.4
Mutual collaboration			
Need for consultation and decision sharing	35.6	4.6	0.9
Need for information exchange	9.1	21.25	3.1
Accountability to and awareness of one’s role among physicians	0.5	0	8.4
Accountability to and awareness of one’s role among patients	3	0	8.4
Each has its role, no more contacts are necessary	2.6	0	0
Better mutual understanding	5	20	0
Knowledge			
Better understanding of the roles and tasks of the three medical professions	8.1	0	0.4
Information on working environment and working conditions	4.1	0	7.9
Information on the legal and regulatory framework	1.2	0	1.3
Evolution of the legal and regulatory framework			
Simplification, harmonisation of working rules	5.8	0	24.7
Strict compliance with the Belgian Privacy Act (patients and physicians’ data)	6.1	3.3	0
Aid to re-integration	1	0	11.4

As shown by the table, the needs and concerns of the three physician groups were different, and the only consensus that emerged at this stage was promoting the availability of clinical data through electronic communication. The GP groups primarily emphasised decision sharing (about the sick leave duration) in mutual collaboration and the availability of clinical data through electronic exchanges. The OPs stressed the communication process and preferred to use the patient as a communication vector rather than electronic messages; they also requested more information exchange. The SIPs were more concerned about an improvement of the legal framework that focuses on the reintegration of sick workers.

### Delphi

Thirty-two experts from the stakeholder groups were invited; twenty-eight experts participated in the first round, and twenty-seven experts participated in the second round (Table 3).

**Table 3:** Participants of the Delphi

Experts	Invited	Participants 1^st^ round	Participants 2^nd^ round	Gender	Age (>50)	Academic affiliation
GPs	12	9	8	4 (on 9) female	6	4
OPs	11	10	10	4 female	7	2
SIPs	9	9	9	2 female	7	1
	32	28	27			

Overall, 9 out of 14 proposals were approved after the first Delphi round. For the second round, the 5 remaining proposals (Q1, Q2, Q7, Q8, and Q9) were rephrased to reach a consensus. Based on the respondents’ written comments, some of the approved proposals (Q6, Q10) were also subject to detailed sub-questions to formalise the proposals.

### Making information transfer operational

As shown in Table 4, a consensus was reached in the first round for only one of the three proposals (the expected positive role of electronic communication in interdisciplinary collaboration - Q.3), and a consensus was reached on two of the four proposals in the second round.

**Table 4:** Delphi consensus levels for “Making information transfer operational”, in % (consensus in bold)

Proposals (round 1)	Global	GPs	OPs	SIPs	Proposals (round 2)	Global	GPs	OPs	SIPs
Q1. Patient’s consent before communication of the SIP’s report to the GP	46.4	66.6	30	44.4	Q1/1. *idem*, even when the patient is registered with his/her doctor's practice	40.7	50	30	44.4
Q2. Directory of contact information of GPs, OPs and SIPs on the Labour Ministry’s website	71.4	**77.8**	70	66.6	Q2/1. Only OPs contact information on the ministry’s website	**76.9**	**100**	60	**77.8**
					Q2/2. SIPs contact information on the NIDHI’s website	**84**	**100**	**80**	**75**
					Q2/3. Identification number for OPs and SIPs to distinguish them from GPs in institutional repositories	69.2	**100**	60	55.6
Q3. Electronic communication → better interdisciplinary collaboration	**85.7**	**77.8**	**90**	**88.9**					

Nevertheless, the proposal of using electronic communication channels to exchange medical information among the physicians was in fact limited by a lack of consensus on the prior informed consent that is to be obtained from the patient (Q1).

*“The patient is the owner of his medical data. We are constantly reminded of this fact in the occupational health field. I don’t understand why it should not be any more of an issue in the case of electronic communication” (OP).*

A particular avoidance process was noted. The availability of contact information in institutional repositories was rejected by the OPs concerning the use of the Ministry of Employment’s website (Q2/1) and was rejected by both the OPs and SIPs regarding the use of a specific identification number on the college of Belgian physicians’ website, which allows them to be distinguished from GPs (Q2/3). The GPs notably reached 100 % consensus for these proposals.

*“It would be very useful, but I am not sure that it is realistic. Furthermore, I am not convinced that this entire medical world is open-minded referring to this modality of total transparency and accessibility of one’s own data” (SIP).*

### Interdisciplinary collaboration

Global consensus was obtained for six of nine proposals in the first round, and the GPs accepted all of these proposals (Q4-Q12) (Table 5). This overall result hides some professional disparities because the OPs and SIPs rejected in the first round some proposals that concerned them (Q4, Q6, Q7, Q8, and Q9). Only one of nine proposals was accepted in the second round, with little support from the GPs (Q10/2), who supported all of the other proposals.

**Table 5:** Delphi consensus levels for “Interdisciplinary collaboration”, in % (consensus in bold)

Proposals (round 1)	Global	GPs	OPs	SIPs	Proposals (round 2)	Global	GPs	OPs	SIPs
Q4. SIP may refer the worker to the OP during sick leave	**82.1**	**88.9**	70	**88.9**					
Q5. GP may refer the worker to the OP during sick leave	**89.3**	**100**	**90**	**77.8**					
Q6. GPs may ask for a copy of the SIP’s decision regarding sick leave	**78.6**	**100**	**80**	55.6	Q6/1. GPs may ask for contact from the SIP if his/her sick note is challenged by the SIP	74.1	**75**	70	**77.8**
Q7. Must the OP transmit the health assessment form to the GP?	60.7	**88.9**	20	**77.8**	Q7/1. Could the … form, showing the OP’s contact information, facilitate communication with the GP?	59.3	**100**	30	55.6
					Q7/2. Must the OP transmit the … form to the GP only when his decision affects employability?	57.7	**75**	40	62.5
Q8. The OP must transmit an excerpt of the list of work hazards to the GP	57.1	**77.8**	50	44.4	Q8/1. An excerpt of the list of work hazards of his/her patient would be useful for the GP	66.7	**75**	50	**77.8**
Q9. The OP must transmit a summary description of the work activity of his/her patient to the GP	70.4	**88.9**	44.4	**77.8**	Q9/1. A summary description of the work activity of his/her patient would be useful to the GP	63	**75**	40	**77.8**
					Q9/2. GPs and OPs should work together to define the content of this summary description	63	**87.5**	70	33.3
Q10. A centralised information summary about occupational risks that were incurred during the patient’s career would be useful	**82.1**	**88.9**	**90**	66.6	Q10/1. A centralised information summary about occupational risks that were incurred during the patient’s career would be useful for the GP	65.4	**85.7**	70	44.4
					Q10/2. Such a […] summary about […] should be set up by public authorities	**76.9**	71.4	**80**	**77.8**
					Q10/3. Such a […] summary about […] should be limited to occupational exposures that are known to cause long-term adverse effects	53.8	**85.7**	30	55.5
Q11. Interdisciplinary collaboration should be part of initial training for all	**85.7**	**77.8**	**90**	**88.9**					
Q12. Interdisciplinary collaboration should be a part of continuous medical education	**100**	**100**	**100**	**100**					

A consensus was reached for the GPs and SIPs to refer workers to the OPs during the sick leave period, regardless of its duration (Q4, Q5). A consensus was also reached in favour of interdisciplinary collaboration by incorporating this topic in both the initial physician training and continuous medical education (Q11, Q12).

The decision sharing between the GPs and SIPs concerning work disability was rejected by the SIPs during the first round (Q6); however, they accepted the decision sharing during the second round provided that it was limited to the cases when the SIPs disputed the GP’s sick note duration (Q6/1).

*“In certain cases, when the SIP anticipates that the GP would not agree with the decision, it is worthwhile, in the limits of possibilities, to communicate and to justify the decision to the GP” (SIP).*

The OPs did not agree to forward some documents to the GPs (i.e., the periodic health assessment form, the list of workplace hazards, and the summary description of the working activity) (Q7, Q8, Q9), regardless of the refinements of the statements that were proposed during the second round (i.e., the facilitation of communication in the cases of a reduction in employability) (Q7/1). This reluctance again suggests an avoidance process. The OPs argued that this sharing would either be unnecessary or infeasible and, in any case, would increase their administrative burden unless the documents could be provided electronically and automatically. Some OPs also stressed the GPs’ lack of competence in correctly interpreting such data.

*“But the question is what the general practitioner will do with these data. He doesn’t have any knowledge of occupational risks. These data seem more interesting for epidemiological research and for the insurance technical files” (OP).*

The proposal of a centralised information summary regarding the occupational risks that are incurred over a worker’s entire career was challenging (Q10). The GPs and OPs considered this summary to be a useful idea; however, the SIPs did not. Moreover, the OPs and SIPs did not agree on this summary’s usefulness to GPs (Q10/1). This summary’s limitation to the exposures that are known to cause long-term adverse health effects was rejected by the OPs and not supported by the SIPs, whereas the GPs strongly agreed with it (Q10/3). The OPs argued regarding the difficulty of retrieving reliable information.

### Knowledge and evolution of a legal and regulatory framework

General proposals for information concerning occupational health and the development of common guidelines for long-term sick leave and reintegration programmes were accepted by the three groups in the first round (Q13/Q14) (Table 6).

**Table 6:** Delphi consensus levels for “Knowledge” and “Evolution of legal and regulatory framework”, in % (consensus in bold)

Proposals (round 1)	Global	GPs	OPs	SIPs
**Knowledge**	**89.3**	**88.9**	**90**	**88.9**
Q13. Information about OP’s mission and risk prevention at work on the Ministry’s website				
**Evolution of the legal and regulatory framework**	**100**	**100**	**100**	**100**
Q14. GPs, OPs and SIPs representatives should develop joint recommendations for long-term sick leave and reintegration programs				

## Discussion

### Main results

From the results, it appears that two areas (reciprocal knowledge and evolution of the legal and regulatory framework) are objects of consensus among the three surveyed medical groups. Information transfer, particularly electronic transfer, was emphasised as an important way to improve. The consensual proposals involving interdisciplinary collaboration indicate specific and practical changes to be implemented when professionals are managing workers who are on sick leave. The collaboration process appeared to be currently more problematic, but the participants correctly identified the need for common training.

### Limitations and strengths

We used a modified consensus design, including a nominal group process in the first round, followed by a classic two-round Delphi process. Finally, consensus was reached on 9/14 proposals in the first Delphi round, and fewer proposals reached consensus in the second round. This result may have been partly attributable to the way that the questions were posed. However, we considered that the lack of consensus on particular proposals reflected some deep misunderstandings or mistrust among the analysed professionals, which could not be explored in this study, and we decided not to perform a third Delphi round.

This study fully respected two central quality criteria for the Delphi technique [25], namely, the short time that elapsed between the two rounds and a very low drop-out rate (1 of 28 participants).

The majority of expert comments were very short, which can be considered a limitation to a deeper comprehension of the problem. However, some defensive reactions among the stakeholder members of the accompanying committee (i.e., “websites containing relevant information do exist” and “a chat box aimed at interdisciplinary communication has just been created”), from both professional bodies and ministries, echoed some of these rather brief expert comments.

Finally, this study explored within a significant number of Belgian stakeholders and field practitioners some interesting proposals for better inter-professional collaboration.

### Reciprocal knowledge

A better understanding of the roles and tasks of the three medical professions and information on the working environment and working conditions seem to remain important aspirations from all the professionals. This desire was emphasised almost two decades ago in Belgium [11, 12]. During this study, we found that few professionals (outside the working environment) were aware of the existence of the Belgian Safe Work Information Centre website [26]. Obviously, more efforts are needed for a better integration of occupational health into the health care system.

### Legal and regulatory framework

The three medical professions agreed on the need for the development of joint recommendations for long-term sick leave and reintegration programmes. It seems obvious that this need should be common work to ensure the consistency and the enforceability of the recommendations [27]. However, a recent report of the Belgian Health Care Knowledge Centre (KCE) indicated that numerous Belgian organisations have published guidelines, with possible gaps or overlap on the same subject, without necessarily reaching the targeted professions; the KCE has strongly recommended multidisciplinary guidelines [28].

### Information transfer

The participants strongly emphasised electronic information transfer. The participants act as if this means of communication will automatically improve collaboration, which is doubtful, whereas the current communication media did not reach this objective. We observe the difficulties that were encountered by the development of the Belgian e-Health system for many years and for various digital applications; one problem at a time must be solved in the various technical, ethical, and sociological domains [29]. Numerous barriers to acceptance and sustainability of e-communication have been described recently abroad [30, 31]. This aspect must be cautiously assessed and carefully implemented to overcome the well-known barriers.

### Interdisciplinary collaboration

Some of the consensual proposals regarding interdisciplinary collaboration that indicate specific and practical changes to be implemented were particularly relevant. These proposals were elaborated with the stakeholders two years before the Ministry of Public Health introduced a bill to organise an interdisciplinary reintegration plan [32]. In this bill, the collaboration of GPs, OPs and SIPs is required, along with other occupational health professionals. In a more practical way, the Belgian scientific societies of GPs, OPs and SIPs launched “trio groups” in the Fall of 2014, where members of the three professions met on and discussed the actual situations that they experienced in daily practice [33].

The participants of the survey requested after the initial training, common training involving interdisciplinary collaboration for all physicians that should be pursued in continuous medical education (CME). Some experiences of these training programmes had interesting results through participatory action, community-based experience or interprofessional-simulation experience [34, 35]. This training is even more important given that stereotyping, common misconceptions and a lack of knowledge concerning other professionals are factors that can impede effective interprofessional collaboration [36].

A recurring finding in the survey was the avoidance process (a type of denial in which some unpleasant aspects of the representation of reality and its meaning are discarded) [37]. This avoidance was observed every time that the health professionals expressed their disagreement on proposals regarding the need for information exchange or shared decision-making. On the one hand, there were constructive comments – even utopian or idealistic – that would permit a possible future consensus among the professionals (i.e., an information exchange that is centred on a return to work, in accordance with the law, and implemented with the use of electronic exchanges). On the other hand, categorical comments presented absolute obstacles. These comments focused on the feasibility of the collaborative process, its usefulness or its unenforceability (i.e., administrative load, workload), and professional competencies. A discrepancy was noted between some proposals (i.e., using the patient as a communication vector) and the reality (this communication channel does not currently work well) [38], as if some health professionals did not want to acknowledge the present lack of collaboration.

The disagreements among the three medical groups question the idea of professional identity itself, which participates in self-recognition through other people. According to Honneth’s theory called “the struggle for recognition”, we identified the following two aspects that relate to moral recognition: (a) compartmentalisation – the proper practices of health professionals are very specific and not easily transferable to other health professionals; and (b) denial – other professionals do not have the ability to understand my language and the guiding proceedings. In the juridical recognition domain, we identified the following two types of behavioural stances: (a) absence – activities that are considered not to belong to the field of other health professionals; and (b) invisibility – the health professional’s specificity is not considered [39].

### Theoretical models for a better understanding of the collaboration process

The avoidance process and the lack of mutual recognition among the practitioners may be better understood if we interpret these observations by using deductive models that are based on sociological theories for collaboration in public health care settings [40, 41]. Although developed on different sociological theories, certain congruencies between these models can be identified. Both models open new avenues to frame collaboration in a broader context.

De Rijk’s Resource Dependence Institutional Cooperation (RDIC) model emphasises the coexistence of the ability and willingness to cooperate and the underlying factors that affect these characteristics, such as dependence, goals, perceptions and resources [40]. We demonstrated a perceived asymmetric dependence between the GPs and SIPs, who both perceived a power difference among other professionals regarding the duration of work incapacity. Because symbiotic dependence is lacking, a powerful actor such as the NIHDI should intervene to favour it. As mentioned by de Rijk, resources that create dependence (i.e., efficient communication channels, the availability of contact information, or common training) are different from the resources that are needed to cooperate, which also depend on motivational factors among professionals. This difference emphasises the importance of combined actions that are initiated at the organisational level in the health care system to improve the capacity to cooperate and actions at an individual level to improve the willingness to cooperate.

D’Amour’s model and typology of collaboration among professionals in health care organisations emphasised the interlinking of the determinants that involve interpersonal relationships (shared goals and vision, and internalisation) and organisational settings (governance and formalisation) [41]. In our study, the three medical practitioners share the goal of facilitating the return of sick workers to work. However, on the internalisation side, the management of interdependencies among the professions and the recognition of one another’s value were strongly lacking. Governance (i.e., taking the leadership role that supports collaboration) and formalisation (i.e., the extent to which procedures are defined, used and assessed [42]) are challenging in the Belgian context. Among the field actors in this study, only the SIPs mentioned these legislative and regulatory concerns.

## Conclusions and recommendations

This study attempted to find a consensus on the improvement of collaboration among GPs, OPs and SIPs when managing sick-listed workers. The results emphasise that the three physician groups are all in agreement regarding several inter-physician collaboration proposals, which were proposed by the researchers to the federal government to be implemented in legislation or were recommendations to resolve unnecessary absences from long-term sickness.

This study also revealed a latent conflict situation among the examined professionals that can arise from a lack of mutual recognition, which we noted as expressing an explicit self-interpretation of the avoidance process that we analysed through theoretical models.

The main conclusion that we can draw from this study is that practical changes and improvements must be included in an extended framework that involves the different determinants of interdisciplinary collaboration that are shown by theoretical models. Collaboration is a product of the actions and behaviours of various partners who need reciprocal knowledge and trust; it also implies political and economic structures that are led by public health authorities.

Considering this global framework allows us to outline some recommendations. It could be worthwhile to organise mixed working groups (i.e., administrative and practitioner representatives) whose objectives would be to define realistic modifications of the working habits of each medical group. Moreover, training involving interdisciplinary collaboration should occur after the initial training of all physicians and during CME.

It could be suitable for the public authority to promote the effective application of new working practices by launching awareness campaigns and adopting new directives, rules or legal dispositions. A better strategy could be for professional and administrative representatives to join existing workshops.

*“Political responsibility is a kind of collective responsibility, and one where the responsibility borne collectively is not dissolvable to the self-conscious collaborative acts of individuals”* [43].

### Ethical approval

The ethics committee of the University Hospital of Liege stated that this study is not covered by the Belgian Human Experimentation Act of May, 7^th^, 2004; the committee has no objections to raise for this study. The participants signed an informed consent form.

## Abbreviations

FPS: Federal Public Service (Belgian Ministry)
GP: general practitioner
NGT: nominal group technique
OHS: occupational health service
IOHS: internal OHS
EOHS: external OHS
OP: occupational health physician
SIP: social insurance physician

## References

[1] Giannakouris K: Ageing characterises the demographic perspectives of the European societies. In: Population and social conditions. edn. Luxembourg: Eurostat Statistics in focus; 2008

[2] Waddel G, Burton AK (2006). Is work good for your health and well-being?.

[3] Schofield RF, Amodeo M (1999). Interdisciplinary teams in health care and human services settings: are they effective?. Health Soc Work.

[4] Cashman S, Reidy P, Cody K, Lemay C (2004). Developing and measuring progress toward collaborative, integrated, interdisciplinary health care teams. J Interprof Care.

[5] San Martin-Rodriguez L, Beaulieu MD, D'Amour D, Ferrada-Videla M (2005). The determinants of successful collaboration: a review of theoretical and empirical studies. J Interprof Care.

[6] Xyrichis A, Lowton K (2008). What fosters or prevents interprofessional teamworking in primary and community care? A literature review. Int J Nurs Stud.

[7] Schwarz B, Neuderth S, Gutenbrunner C, Bethge M (2015). Multiprofessional teamwork in work-related medical rehabilitation for patients with chronic musculoskeletal disorders. Journal of rehabilitation medicine.

[8] Anema JR, Van Der Giezen AM, Buijs PC, Van Mechelen W (2002). Ineffective disability management by doctors is an obstacle for return-to-work: a cohort study on low back pain patients sicklisted for 3–4 months. Occup Environ Med.

[9] Mortelmans K, Donceel P, Lahaye D (2006). Disability management through positive intervention in stakeholders' information asymmetry. A pilot study. Occupational medicine (Oxford, England).

[10] Mortelmans K (2006). Enhancing work resumption of patients on sub-acute sickness absence. Intervening in information asymmetry among medical stakeholders involved in disability management in Belgium.

[11] Donceel P (1999). De bijdrage van de verzekeringsgeneeskundige tot de professionele reïntegratie na een heelkundige behandeling van de lumbale wervelzuil.

[12] De Graeve D, Maes R, Dierckx G, A D: Ziekteverzuim en het toekennen van arbeidsongeschiktheid. In*.* Brussel: Federale Diensten voor Wetenschappelijke, Technische en Culturele Aangelegenheden; 1995

[13] Mortelmans K, Masschelein R, Moens G (2004). Samenwerking tussen de bedrijfsarts, verzekeringsarts en curatieve arts. Resultaten van een cross-sectioneel onderzoek bij Belgische bedrijfsartsen. Tijdschrift voor Gezondheidswetenschappen.

[14] Van de Ven AH, Delbecq AL (1972). The nominal group as a research instrument for exploratory health studies. Am J Public Health.

[15] Dalkey NC (1969). The Delphi method: an experimental study of group opinion.

[16] Davies S, Romano PS, Schmidt EM, Schultz E, Geppert JJ, McDonald KM (2011). Assessment of a novel hybrid Delphi and Nominal Groups technique to evaluate quality indicators. Health Serv Res.

[17] Kadam UT, Jordan K, Croft PR (2006). A comparison of two consensus methods for classifying morbidities in a single professional group showed the same outcomes. J Clin Epidemiol.

[18] Landeta J, Barrutia J, Aitziber L (2011). Hybrid Delphi: A methodology to facilitate contribution from experts in professional contexts. Technological Forecasting and Social Change.

[19] Fink A, Kosecoff J, Chassin M, Brook RH (1984). Consensus methods: characteristics and guidelines for use. Am J Public Health.

[20] Hsu C-C, Sandford BA (2007). The Delphi technique: making sense of consensus. Practical Assessment, Research and Evaluation.

[21] Rayens MK, Hahn EJ (2000). Building consensus using the policy delphi method. Policy, Politics, and Nursing Practice.

[22] Farmer T, Robinson K, Elliott SJ, Eyles J (2006). Developing and implementing a triangulation protocol for qualitative health research. Qual Health Res.

[23] Corbin J, Strauss A (2007). Basics of Qualitative Research. Techniques and Procedures for Developing a Grounded Theory, 3rd edn.

[24] Fetterman DM (2010). Ethnography.

[25] Bardecki MJ (1984). Participants' response to the Delphi method: An attitudinal perspective. Technological Forecasting and Social Change.

[26] Belgian Safe Work Information Center (BeSWIC) [www.beswic.be/fr ]

[27] Yassi A, Ostry AS, Spiegel J, Walsh G, de Boer HM (2002). A collaborative evidence-based approach to making healthcare a healthier place to work. Hosp Q.

[28] Desomer A, Dilles T, Steckel S, Duchesnes C, Vanmeerbeek M, Peremans L (2013). Dissemination and implementation of clinical practice guidelines in Belgium. Health Services Research (HSR).

[29] Storms H, Marquet K, Nelissen K, Hulshagen L, Lenie J, Remmen R (2014). Implementing an electronic medication overview in Belgium. BMC research notes.

[30] Rudin RS, Motala A, Goldzweig CL, Shekelle PG (2014). Usage and effect of health information exchange: a systematic review. Ann Intern Med.

[31] Kierkegaard P, Kaushal R, Vest JR (2014). How could health information exchange better meet the needs of care practitioners?. Applied clinical informatics.

[32] Réintégration des personnes en incapacité de travail dans la loi-programme 2014 [http://www.beswic.be/fr/news_board/reintegration]

[33] Santé et bien-être au travail [http://www.ssmg.be/cellules-specifiques/sante-et-bien-etre-au-travail]

[34] Vingilis E, Forchuk C, Orchard C, Shaw L, King G, McWilliam C, Khalili H, Edwards B, Osaka W: Development, Implementation, and Formative Evaluation of Pre-licensure Workshops Using Participatory Action Research to Facilitate Interprofessional, Client-Centred Mental Healthcare. Journal of research in Interprofessional Practice and education 2011, 2.1(July). http://jripe.org/jripe/index.php/journal/article/viewArticle/37.

[35] Bridges DR, Davidson RA, Odegard PS, Maki IV, Tomkowiak J. Interprofessional collaboration: three best practice models of interprofessional education. Medical education online. 2011;16. 10.3402/meo.v16i0.6035 PMC3081249 21519399

[36] Cook K, Stoecker J: Healthcare Student Stereotypes: A Systematic Review with Implications for Interprofessional Collaboration. Journal of research in Interprofessional Practice and education 2014, 4.2(November). http://www.jripe.org/index.php/journal/article/viewArticle/151.

[37] Fassin D: Du déni à la dénégation. Psychologie politique de la représentation des discriminations. In: De la question sociale à la question raciale ? Edited by Fassin D, Fassin E. Paris: La Découverte; 2006

[38] Mairiaux P, Vanmeerbeek M, Schippers N, Denoël P, Tiedtke C, Mortelmans K, et al: Collaboration improvement between general practitioners, occupational physicians and insurance medical advisors for a better management of occupationnal diseases. In*.* Brussels: Federal Public Service Employment, Labour and Social Dialogue; 2011

[39] Honneth A (1995). The struggle for recognition. The moral grammar of social conflicts.

[40] de Rijk A, van Raak A, van der Made J (2007). A new theoretical model for cooperation in public health settings: the RDIC model. Qual Health Res.

[41] D'Amour D, Goulet L, Labadie JF, Martin-Rodriguez LS, Pineault R (2008). A model and typology of collaboration between professionals in healthcare organizations. BMC Health Serv Res.

[42] Bodewes WEJ (2002). Formalization and innovation revisited. European Journal of Innovation Management.

[43] Young IM (2004). Responsibility and Global Labor Justice. Journal of Political Philosophy.

